# *In vitro* effects of apigenin, curcumin, and fumaric acid on intracellular ROS and TNF-α responses in *Escherichia coli*-stimulated canine PBMCs

**DOI:** 10.3389/fvets.2026.1930766

**Published:** 2026-09-09

**Authors:** Alma V. Móritz, Beatrix Puskás, Noémi Nagy, Ákos Jerzsele, Orsolya Farkas

**Affiliations:** 1 Department of Pharmacology and Toxicology, University of Veterinary Medicine, Budapest, Hungary; 2 National Laboratory of Infectious Animal Diseases, Antimicrobial Resistance, Veterinary Public Health and Food Chain Safety, University of Veterinary Medicine, Budapest, Hungary

**Keywords:** apigenin, canine PBMCs, curcumin, endotoxemia, *Escherichia coli*, fumaric acid

## Abstract

**Background:**

Chronic inflammatory enteropathy (CIE) is a common gastrointestinal disorder in dogs and is associated with dysregulated inflammatory and oxidative responses, potentially contributing to the development of endotoxemia.

**Methods:**

The present study investigated the effects of apigenin, curcumin, and fumaric acid on inflammatory and oxidative responses in canine peripheral blood mononuclear cells (PBMCs) under endotoxin- and bacteria-associated stimulation. PBMCs obtained from three healthy donor dogs were exposed to *Escherichia coli* O111:B4 or O127:B8 lipopolysaccharide (LPS), or to enrofloxacin-treated *E. coli*, and subsequently treated with the tested compounds. Cellular metabolic activity, LDH release, intracellular reactive oxygen species (ROS), H_2_O_2_ production, and TNF-α secretion were assessed. Minimum inhibitory concentration (MIC), mutant prevention concentration (MPC), and fractional inhibitory concentration (FIC) indices were determined to evaluate antimicrobial activity and interactions with enrofloxacin, amoxicillin–clavulanic acid, and gentamicin.

**Results:**

None of the tested compounds significantly altered metabolic activity or LDH release under the applied conditions. Curcumin consistently reduced intracellular ROS and H_2_O_2_ production under selected stimulated conditions and decreased TNF-α secretion. Apigenin increased DCF-derived intracellular ROS under basal conditions and following lower-intensity O111 LPS stimulation, while reducing H_2_O_2_ and TNF-α production under several conditions, indicating a stimulus- and assay-dependent effect on cellular redox responses. Fumaric acid showed more limited, stimulus-dependent effects on oxidative and inflammatory parameters. Antimicrobial activity was also evaluated against four canine septicemic *E. coli* isolates. Due to limited solubility, the MIC and MPC values of the tested compounds were ≥1,024 μg/mL and could not be reliably determined above this concentration. Curcumin showed synergistic interaction with amoxicillin–clavulanic acid only against isolate No. 863, whereas fumaric acid exhibited concentration-dependent antagonism with enrofloxacin.

**Conclusion:**

Overall, the findings demonstrate compound- and stimulus-dependent modulation of oxidative and inflammatory responses in canine PBMCs and support further investigation of these compounds in disease-relevant models of endotoxemia and canine CIE.

## 1 Introduction

Chronic inflammatory enteropathies (CIE) in dogs are characterized by persistent or recurrent gastrointestinal signs—such as diarrhea, vomiting, and weight loss lasting at least 3 weeks—combined with histopathologic evidence of mucosal inflammation, following the exclusion of other etiologies (1, 2). These disorders represent a substantial proportion of referral cases in small animal practice and remain clinically challenging due to their heterogeneous presentation and multifactorial pathogenesis (3). The development of CIE involves a complex interplay between host genetic susceptibility, environmental influences (including diet), and alterations in the intestinal microbiota. A key pathological feature is the disruption of intestinal epithelial barrier integrity, which normally serves as a critical interface between luminal contents and the host immune system. Barrier dysfunction facilitates the translocation of microbial components, particularly lipopolysaccharides (LPS), into the systemic circulation, contributing to endotoxemia and sustained immune activation (4). LPS, a major structural component of Gram-negative bacterial outer membranes, plays a pivotal role in driving inflammatory responses through activation of the toll-like receptor 4 (TLR4) signaling pathway. This interaction triggers downstream activation of nuclear factor-kappa B (NF-κB) and mitogen-activated protein kinase (MAPK) cascades, leading to the production of proinflammatory cytokines such as tumor necrosis factor alpha (TNF-α) and interleukin-1 beta (IL-1β), as well as increased generation of reactive oxygen species (ROS) (5). While these responses are essential for host defense, their chronic activation contributes to oxidative stress, tissue damage, and perpetuation of inflammation. In addition to local intestinal effects, systemic consequences of chronic inflammation and endotoxemia have been increasingly recognized, including associations with extraintestinal conditions such as renal, cardiovascular, and neurological disorders (6). Circulating immune cells, particularly peripheral blood mononuclear cells (PBMCs), play a central role in mediating these systemic inflammatory responses. Therefore, *in vitro* PBMC models stimulated with bacterial components or whole bacteria may provide a valuable and physiologically relevant approach for modeling aspects of the inflammatory response associated with endotoxemia and for investigating inflammation- and oxidative stress-related mechanisms potentially involved in CIE. The intestinal microbiome itself is a key regulator of both metabolic and immune homeostasis. Dysbiosis, characterized by reduced microbial diversity and overrepresentation of pathogenic or opportunistic bacteria, is a hallmark of CIE (7). Increased abundance of Gram-negative species such as *E. coli* has been reported. These bacteria contribute to disease progression through LPS production, activation of inflammatory signaling pathways, and induction of oxidative stress.

In recent years, plant-derived and naturally occurring bioactive compounds have gained considerable attention as potential supportive agents in inflammatory diseases. These compounds have been reported to exhibit a combination of antioxidant, anti-inflammatory, and antimicrobial properties, making them attractive candidates for integrative therapeutic approaches (8, 9). Oxidative stress plays a central role in the pathophysiology of CIE, as excessive ROS production contributes to epithelial damage, increased intestinal permeability, and amplification of inflammatory signaling (10). Therefore, compounds capable of modulating redox balance while simultaneously influencing immune responses may provide dual benefits in the management of chronic enteropathies. Among the wide range of bioactive molecules, curcumin, apigenin, and fumaric acid have emerged as particularly promising candidates due to their well-documented molecular effects and relative safety profiles (11–14).

Curcumin, a polyphenolic diketone derived from the rhizome of *Curcuma longa*, has been extensively studied for its pleiotropic biological activities. Its anti-inflammatory effects are primarily mediated through inhibition of key signaling pathways, including NF-κB and MAPK, as well as suppression of TLR4-mediated responses. These mechanisms result in reduced expression of proinflammatory cytokines such as TNF-α, IL-1β, and IL-6 (5). In addition to its immunomodulatory properties, curcumin has been reported to reduce intracellular ROS production and modulate antioxidant signaling pathways. It functions both as a direct scavenger of reactive oxygen and nitrogen species and as an inducer of endogenous antioxidant defense systems, including superoxide dismutase (SOD), catalase (CAT), and glutathione peroxidase (GPx). This dual mode of action is particularly relevant in conditions characterized by chronic oxidative stress, such as CIE. Curcumin has also demonstrated antimicrobial effects against a broad range of bacterial species, including Gram-negative pathogens. Although its direct antibacterial potency is generally lower than that of conventional antibiotics, it may enhance antimicrobial efficacy through mechanisms such as disruption of bacterial membranes, inhibition of quorum sensing, and modulation of efflux pump activity (15). These properties suggest a potential role as an adjunct to antibiotic therapy.

Apigenin is a naturally occurring flavone widely distributed in plant-based foods such as parsley, celery, and chamomile. It has attracted significant interest due to its diverse pharmacological activities, including antioxidant, anti-inflammatory, and antimicrobial effects (16–18). At the molecular level, apigenin modulates several inflammatory pathways. It inhibits the expression of cyclooxygenase-2 (COX-2) and inducible nitric oxide synthase (iNOS), suppresses NF-κB activation, and interferes with MAPK signaling, thereby reducing the production of proinflammatory mediators (19–23). In addition, apigenin contributes to the regulation of oxidative stress by scavenging free radicals and influencing cellular redox balance. Intestinal barrier function and modulate gut microbiota in other experimental models (24). Furthermore, it has demonstrated antimicrobial activity against *E. coli*, potentially through disruption of membrane integrity and interference with nucleic acid synthesis (25, 26). However, its effects may vary depending on the cellular and environmental context, highlighting the importance of investigating its activity under specific experimental conditions.

Fumaric acid (FA) is a naturally occurring dicarboxylic acid and an intermediate of the tricarboxylic acid (TCA) cycle. Dietary supplementation with fumaric acid has been investigated in animal studies, including broiler chickens, where it has been associated with effects on growth performance, immune responses, and antioxidant status (27). Although fumaric acid and dimethyl fumarate (DMF) are chemically distinct compounds, the latter has been more extensively investigated in human medicine for its anti-inflammatory and antioxidant effects, particularly in the treatment of psoriasis and multiple sclerosis (28). Fumaric acid has been investigated for its potential effects on cellular redox balance and inflammatory processes, although the underlying mechanisms remain less well characterized than those of its derivative, dimethyl fumarate. In particular, activation of the Nrf2 pathway and subsequent modulation of antioxidant and inflammatory responses have been extensively described for dimethyl fumarate (29, 30). However, these findings should not be directly extrapolated to fumaric acid, as the two compounds differ in their chemical structure and biological activity. In the context of gastrointestinal health, fumaric acid has been used as a dietary acidifier in livestock production, where it contributes to improved growth performance, modulation of gut microbiota, and inhibition of pathogenic bacteria such as *E. coli* (31, 32). Its antimicrobial effects are thought to involve disruption of bacterial energy metabolism and membrane potential. Although data in canine models are limited, these properties may warrant further investigation of its potential role as an adjunctive intervention in intestinal disorders.

Taken together, these compounds may contribute to the preservation of intestinal and systemic immune homeostasis by reducing oxidative stress, modulating inflammatory signaling, and potentially influencing bacterial viability. Importantly, their combined use with conventional antibiotics may offer a strategy to enhance therapeutic efficacy while mitigating the risks associated with antimicrobial resistance.

Two commonly used *E. coli* LPS serotypes (O111:B4 and O127:B8) were included because LPS preparations from different serotypes may differ in their biological potency and in the magnitude of TLR4-mediated cellular responses due to structural variation in the lipid A and O-antigen regions. Using two LPS serotypes allowed us to assess whether the observed effects of the tested compounds were reproducible across different endotoxin stimuli rather than being specific to a single commercial LPS preparation. To further increase the biological relevance of the model, PBMCs were also stimulated with an antibiotic-inactivated canine clinical *E. coli* isolate, providing a more complex bacterial stimulus than purified LPS alone.

In the present study, we evaluated the effects of curcumin, apigenin and fumaric acid on PBMC metabolic activity, intracellular ROS production, TNF-α release and antibacterial activity. Specifically, we investigated their effects on metabolic activity, cytotoxicity, intracellular ROS production, and TNF-α release in peripheral blood mononuclear cells (PBMCs) stimulated with LPS and *E. coli*. In addition, we assessed their antimicrobial activity by determining minimum inhibitory concentration (MIC) and mutant prevention concentration (MPC) values against clinical canine *E. coli* isolate 863 and examined potential interactions with amoxicillin–clavulanic acid, gentamicin and enrofloxacin using fractional inhibitory concentration (FIC) analysis. By integrating cellular, biochemical, and microbiological approaches, this study aims to provide a comprehensive evaluation of these natural compounds and their potential relevance to inflammatory and oxidative processes associated with CIE, with particular attention to mechanisms that may be associated with the development of endotoxemia.

## 2 Materials and methods

### 2.1 Isolation and culture of white blood cells

The mononuclear cells used in the experiment were isolated from blood tissue. Blood samples were collected with the owners’ consent from three healthy dogs (one PBMC isolation per donor) that were regularly vaccinated and dewormed. During the study, the Histopaque density gradient centrifugation technique was used for cell separation, which is a rapid and simple method based on density gradient centrifugation. The isolation procedure was carried out according to the manufacturer’s instructions. Blood was collected into EDTA tubes, and 3 mL of anticoagulated whole blood was carefully layered onto 3 mL of Histopaque 1.077 (Merck, Darmstadt, Germany). Centrifugation was performed at 400 × g for 30 min at room temperature. Due to differences in cell density, distinct layers formed after centrifugation. This enabled the separation of the required white blood cells, which were located at the interface between the Histopaque solution and the plasma. The separated fractions were transferred into new centrifuge tubes, and 1 mL of Red Blood Cell lysis solution (Roche Diagnostics, Mannheim, Germany) was added. Samples were then brought up to 10 mL with phosphate-buffered saline (PBS; Gibco, London, UK) and centrifuged again at 400 × g for 7 min at room temperature. Repeating this process ensured complete removal of red blood cells. After purification, samples were washed twice with 10 mL PBS and centrifuged at 250 × g for 10 min at room temperature (33). The supernatant was discarded and the cells were resuspended in culture medium.

Viable cells were counted using a Bürker chamber with trypan blue staining (Sigma-Aldrich, Darmstadt, Germany). The cell suspension was adjusted to a density of 2 × 10^5^ cells/mL and seeded into 24- and 96-well plates. Cells were cultured in RPMI-1640 medium (Roswell Park Memorial Institute medium; ThermoFisher, Rockford, USA) containing L-glutamate and sodium bicarbonate, supplemented with 10% fetal bovine serum (FBS; EuroClone, Pero, Italy) and 1% PenStrep (Lonza, Verviers, Belgium). Penicillin–streptomycin remained present throughout all PBMC experiments, including bacterial stimulation. Incubation was carried out for 24 h at 37 °C in a humidified incubator with 5% CO₂ and 95% relative humidity. Oxidative and inflammatory responses were induced by treatment with *E. coli* O111 or O127 LPS at a concentration of 1 μg/mL. Alternatively, cultures were exposed to a canine septicemic *E. coli* isolate (No. 863), recovered in 2021 by the Department of Microbiology and Infectious Diseases of the University of Veterinary Medicine, Budapest. The bacterial inoculum was diluted to 10^3^ CFU/mL before being applied to the cell cultures, this corresponded to a bacteria-to-cell ratio of 1:200. Preliminary experiments demonstrated that the standard PenStrep concentration used for PBMC culture was insufficient to prevent bacterial proliferation of the clinical *E. coli* isolate therefore, the bacterial suspension was treated with enrofloxacin (0.5 μg/mL) for 24 h before PBMC stimulation. Complete bacterial inactivation was confirmed by the absence of colony growth following agar plating. The treated bacteria were subsequently used as a non-viable bacterial stimulus (34). Enrofloxacin-only controls were included during model validation to assess potential effects of residual antibiotic on PBMC responses. The effects of apigenin, fumaric acid, and curcumin were subsequently evaluated by assessing intracellular ROS and extracellular H_2_O_2_ production, as well as TNF-α levels, to determine their potential anti-inflammatory and oxidative stress–modulating effects. Curcumin (≥94%, Sigma-Aldrich, Darmstadt, Germany) was applied at concentrations of 6.25 and 25 μg/mL, while apigenin (≥95%, Sigma-Aldrich, Darmstadt, Germany) and fumaric acid (≥99%, ThermoFisher, Rockford, USA) were used at 12.5 and 50 μg/mL. The compounds were dissolved in DMSO, resulting in a final DMSO concentration of 1% (v/v) in the treatment solutions. All DMSO stock solutions and final treatment solutions were freshly prepared immediately before each experiment to minimize potential compound degradation and ensure solution stability. At the concentrations used, the treatment solutions maintained a neutral pH. Preliminary experiments confirmed that 1% DMSO did not exert detectable effects under the experimental conditions. The selected concentrations of LPS, bacterium and antioxidants were based on previous experiments and on data from the scientific literature (16, 19, 34–37). After removing the culture medium, the treatment solutions were added to the experimental samples, and experiments were performed after 24 h of incubation.

Control cells received only culture medium and were compared with samples treated exclusively with *E. coli* or compound treatments. Data from cells treated with both *E. coli* and tested compounds were compared to those treated with *E. coli* alone.

Because several of the investigated compounds are intrinsically colored and the applied assays rely on colorimetric or fluorometric detection, additional control measurements were performed to exclude potential interference of the test compounds with the analytical readouts. For this purpose, cell-free assay controls containing the respective test compounds in cell-free culture medium and the corresponding assay reagents were prepared and analysed under the same experimental conditions as the samples. Background signals generated by the compounds themselves, the culture medium, or interactions between the compounds and the assay reagents were assessed and compared with the signals obtained from the experimental samples. For each assay, appropriate reagent and sample controls were included to determine the background signal in the absence of cells or biological analytes. Where applicable, compound-only controls were also included to assess intrinsic absorbance or fluorescence of apigenin, curcumin, and fumaric acid at the wavelengths used for detection. Interference controls were performed independently for the different analytical assays, using the same concentrations of the tested compounds and the same assay conditions as applied to the corresponding experimental measurements.

### 2.2 Assessment of metabolic activity and cytotoxicity

In our study, a CCK-8 (Cell Counting Kit-8; Sigma-Aldrich, Darmstadt, Germany) assay was used to evaluate *in vitro* metabolic activity (38). The CCK-8 assay is a highly sensitive, colorimetric, and reproducible method. The reagent contains a water-soluble tetrazolium salt (WST-8), which is reduced by cellular dehydrogenase enzymes to an orange-colored formazan product in the presence of an electron mediator (usually NADH) (39). The intensity of the color produced reflects the metabolic activity of viable cells. The assay was performed according to the manufacturer’s instructions. Ten microliters of CCK-8 solution were added to each well containing PBMC cells and incubated in a 96-well plate at 37 °C with 5% CO₂ for 1 h. Absorbance was measured at 450 nm using a microplate reader (SpectraMax iD3, Molecular Devices, San Jose, CA, USA). Absorbance values were converted to percentages relative to the control group.

The impact of curcumin, apigenin, and fumaric acid treatments on PBMC viability was evaluated by determining lactate dehydrogenase (LDH) activity in the culture supernatants using a commercially available LDH Activity Assay Kit (Sigma-Aldrich, Darmstadt, Germany), following the manufacturer’s protocol. This method is based on the quantification of LDH released into the extracellular medium as a consequence of compromised cell membrane integrity (40, 41). LDH is a stable intracellular enzyme that is rapidly released upon cellular damage or lysis; therefore, its extracellular concentration serves as a reliable indicator of cytotoxicity. For the measurement, 10 μL of cell culture supernatant from each well was transferred into a 96-well plate. A freshly prepared reaction mixture, containing lactate as substrate, NAD^+^ as cofactor, and a colorimetric detection system, was added to each sample (100 μL per well). In this coupled enzymatic reaction, LDH catalyzes the conversion of lactate to pyruvate, accompanied by the reduction of NAD^+^, which subsequently leads to the formation of a colored formazan product. Following reagent addition, the plate was incubated at 37 °C for 30 min in the dark. Absorbance was then recorded at 450 nm using a microplate reader (SpectraMax iD3, Molecular Devices, San Jose, CA, USA). Cytotoxicity was determined by comparing LDH activity in treated samples to that of untreated controls and was expressed either as percentage cell death or as fold change relative to the control group.

### 2.3 Determination of intracellular ROS levels

To evaluate the redox balance of cells and detect ROS levels, the widely used DCFH-DA assay (2′,7′-dichlorofluorescein diacetate; Sigma-Aldrich, Darmstadt, Germany) was applied. DCFH-DA is a non-fluorescent, cell-permeable molecule and a precursor of DCF (2′,7′-dichlorofluorescein). Intracellular esterases cleave DCFH-DA to form H₂DCF (2′,7′-dihydrodichlorofluorescein), which, due to its polarity, becomes trapped inside the cell. This compound is subsequently oxidized by ROS, forming fluorescent DCF, thereby providing information about intracellular ROS levels (42). The resulting fluorescence provides a relative measure of intracellular oxidative activity.

Due to the photosensitivity of DCFH-DA, the assay was conducted in the dark. A stock solution was prepared by dissolving the powdered DCFH-DA in dimethyl sulfoxide, followed by dilution with plain culture medium to prepare the working solution in a 50 mL centrifuge tube. After washing the cells with PBS in a 24-well plate, the DCFH-DA working solution was added and incubated for 1 h at 37 °C. The solution was then removed, and 200 μL M-PER (Mammalian Protein Extraction Reagent; ThermoFisher, Rockford, USA) lysis buffer was added to each well. Samples were left for 10 min to ensure complete cell lysis, then centrifuged at 4500 rpm for 5 min at 4 °C. From the supernatant, 100 μL was transferred to a 96-well microtiter plate. Fluorescence intensity was measured at excitation 480 nm and emission 530 nm using a SpectraMax iD3 spectrophotometer (Molecular Devices, San Jose, CA, USA). Values were expressed as percentages relative to the control.

### 2.4 Determination of extracellular H_2_O_2_ levels

H_2_O_2_ production was quantified using the Amplex Red Hydrogen Peroxide/Peroxidase Assay Kit (ThermoFisher, Rockford, USA) according to the manufacturer’s instructions. The assay is based on the oxidation of Amplex Red (10-acetyl-3,7-dihydroxyphenoxazine) by H_2_O_2_ in the presence of horseradish peroxidase (HRP), resulting in the formation of the fluorescent product resorufin. Resorufin exhibits excitation and emission maxima at approximately 571 and 585 nm, respectively, allowing quantitative determination of H_2_O_2_ levels by fluorescence measurement.

Briefly, following the respective experimental treatments, cell culture samples were collected and used for the determination of H_2_O_2_ production. The Amplex Red reagent was dissolved in anhydrous DMSO and protected from light. A working reaction solution was prepared using the supplied 5 × reaction buffer, Amplex Red reagent, and HRP, according to the manufacturer’s recommended concentrations. The assay was performed in a 96-well microplate. A series of H_2_O_2_ standards was prepared in the assay reaction buffer to generate a standard curve for the quantitative determination of H_2_O_2_ concentrations. Appropriate reagent blanks and untreated/control samples were included in each assay to account for background fluorescence and basal H_2_O_2_ levels.

Following addition of the reaction mixture, the microplate was incubated for 30 min at room temperature and protected from light. Fluorescence was subsequently measured using a fluorescence microplate reader at an excitation wavelength of 540 nm and an emission wavelength of 590 nm. H_2_O_2_ concentracions were calculated from the standard curve after subtraction of the corresponding background fluorescence and subsequently expressed as percentages relative to the corresponding untreated control.

### 2.5 Determination of TNF-α levels

The enzyme-linked immunosorbent assay (ELISA) is a widely used serological method based on antigen–antibody interactions and performed in a solid phase. ELISA is generally efficient, rapid, and highly sensitive and specific (43). TNF-α is a multifunctional cytokine produced during inflammation and regulates various cellular responses by binding to TNF-1 or TNF-2 receptors (44). In this study, cytokine levels induced by LPS or *E. coli* bacteria were determined using a canine TNF-α sandwich ELISA kit (ElabScience Bionovation Inc., Texas, USA), following the manufacturer’s instructions. After 24 h of treatment with LPS and compound treatments, supernatants were collected and absorbance was measured at 450 nm. Data were converted to concentration values (pg/mL) and expressed as percentages relative to control.

In addition to TNF-α, canine IL-1β, IL-2, and IL-6 were also assessed using the corresponding canine ELISA kits (ElabScience Bionovation Inc., Texas, USA) following the manufacturers’ instructions. However, the measured concentrations for these cytokines were below the quantifiable range of the respective calibration curves and therefore could not be reliably quantified or included in the statistical analysis. Consequently, only TNF-α data were considered for further analysis.

### 2.6 Statistical analysis

All statistical analyses were performed using R 3.3.2 (2016) software (R Foundation, Vienna, Austria). Three independent biological replicates were included in each experimental group, corresponding to three individual dogs. For each dog, four technical replicate measurements were performed, resulting in a total of 12 technical measurements per experimental condition. To avoid pseudoreplication, the four technical replicates obtained from each dog were averaged, and the resulting mean value was used as a single biological observation for statistical analysis. Thus, the individual dog was considered the experimental unit, with *n* = 3 biological replicates per experimental group.

Data are presented as mean ± standard deviation (SD). Differences among experimental groups were assessed using one-way analysis of variance (one-way ANOVA) using the *post hoc* Tukey test for multiple comparisons. Prior to statistical analysis, the data were evaluated for the assumptions underlying ANOVA, including the approximate normality of the data distribution and homogeneity of variances. Statistical significance was defined as *p* < 0.05. The following significance levels were used: *p* < 0.05 (^*^), *p* < 0.01 (^**^), and *p* < 0.001 (^***^), whereas *p* ≥ 0.05 was considered not statistically significant.

### 2.7 Antimicrobial activity studies

Minimum inhibitory concentration (MIC) is defined as the lowest concentration of an antimicrobial agent that prevents visible growth of a microorganism following incubation. Determination of the MIC provides information on bacterial susceptibility and the antimicrobial activity of the tested compounds. MIC values can be determined using agar-based or broth-based methods; in broth microdilution assays, serial twofold dilutions of the tested compounds are prepared in microtiter plates and bacterial growth is assessed following incubation, typically for 18–24 h.

For the present study, MIC, mutant prevention concentration (MPC), and checkerboard assays were performed using four canine septicemic *E. coli* isolates (No. 340, 404, 863, and 865). All isolates were recovered from cases of canine septicemia and originated from the bacterial collection of the Department of Microbiology and Infectious Diseases, University of Veterinary Medicine Budapest. Isolates No. 340 and 404 were recovered in 2020, whereas isolates No. 863 and 865 were recovered in 2021. The No. 863 isolate was also used in the subsequent cellular experiments.

For MIC determination, twofold serial dilutions of apigenin, curcumin, and fumaric acid were prepared in Mueller–Hinton broth. Bacterial suspensions were adjusted to a 0.5 McFarland standard and subsequently diluted 200-fold before inoculation. The bacterial suspensions were inoculated into the prepared microtiter plates, and the cultures were incubated at 37 °C for 18 h. Following incubation, the MIC was determined as the lowest concentration of the tested compound at which no visible bacterial growth was observed.

MPC values were subsequently determined to estimate the concentration required to inhibit the growth of the least susceptible bacterial subpopulation. For MPC determination, a higher bacterial inoculum corresponding to a 1.0 McFarland standard was used. The plate preparation and compound dilution procedure were otherwise performed as described for the MIC assay. Following inoculation, the plates were incubated at 37 °C for 72 h, after which bacterial growth was assessed and the MPC was determined as the lowest concentration at which no visible growth was detected.

### 2.8 Synergy testing

The potential synergistic effects of apigenin, curcumin, and fumaric acid in combination with amoxicillin–clavulanic acid, enrofloxacin, and gentamicin were investigated against the four canine septicemic *E. coli* isolates. Synergism was defined as an enhanced antimicrobial effect of the combination compared with the activity of either compound used alone. Checkerboard assays were performed using twofold serial dilutions of the tested compounds and antibiotics based on their previously determined MIC values.

Bacterial inocula were prepared and adjusted to a final concentration of 5 × 10^5^ CFU/mL in Mueller–Hinton broth. Serial twofold dilutions of the tested compounds and antibiotics were prepared, and 45 μL of the antibiotic dilution was transferred to the corresponding wells containing the tested compound dilution series. Subsequently, 10 μL of the bacterial suspension was added to each well, except for the negative control wells. The plates were incubated at 37 °C for 24 h. Following incubation, bacterial growth was assessed, and the MIC values of the individual agents and their combinations were determined.

The fractional inhibitory concentration (FIC) of each tested compound was calculated by dividing its MIC in combination with the antibiotic by its MIC when tested alone. Similarly, the FIC of each antibiotic was calculated by dividing its MIC in combination by its MIC when tested alone. The FIC index (FICI) was calculated as the sum of the individual FIC values:

$$\begin{array}{l} {\mathrm{FIC}}_{\text{tested compound}}={\mathrm{MIC}}_{\text{tested compound in combination}}/\\ {\mathrm{MIC}}_{\text{tested compound alone}}. \end{array}$$

$${\mathrm{FIC}}_{\text{antibiotic}}={\mathrm{MIC}}_{\text{antibiotic in combination}}/{\mathrm{MIC}}_{\text{antibiotic alone}}.$$

$${\mathrm{FIC}}_{\text{index}}={\mathrm{FIC}}_{\text{tested compound}}+{\mathrm{FIC}}_{\text{antibiotic}}.$$

If the FIC index is <0.5, synergism is present. Values between 0.5 and 1 indicate partial synergism or additive effects. Values between 1 and 4 indicate no interaction, while values >4 suggest antagonism.

## 3 Results

### 3.1 Assessment of metabolic activity

**Figure 1:**
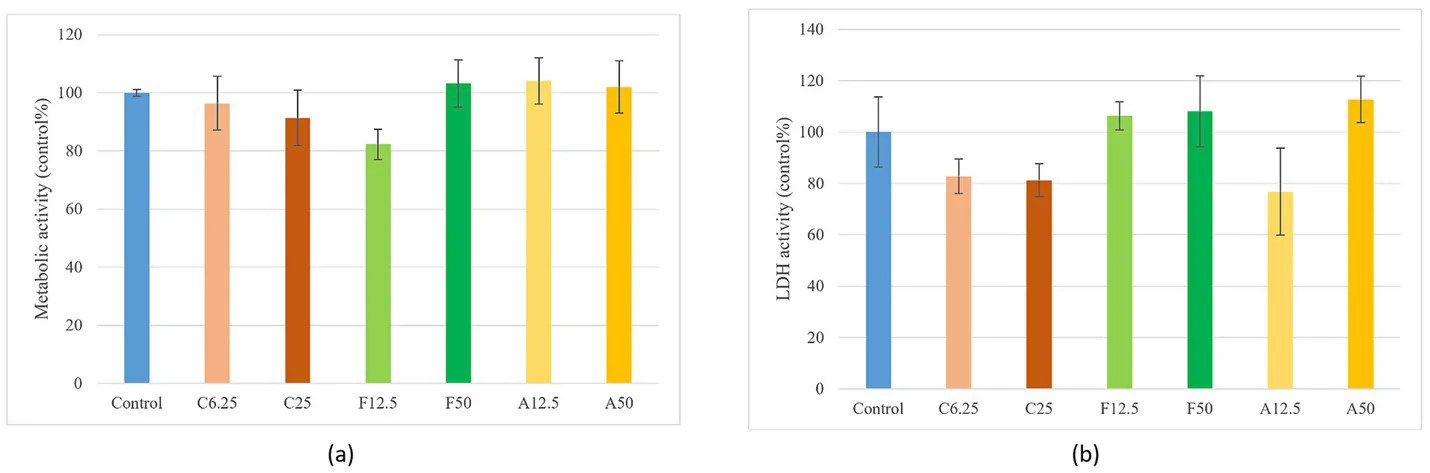
Effects of curcumin, fumaric acid, and apigenin on metabolic activity and cytotoxicity in canine PBMC cultures. **(a)** Cell metabolic activity determined by CCK-8 assay. **(b)** Cytotoxicity assessed by LDH release. Data are presented as mean ± standard deviation (SD), with the control group set to 100% (*n* = 3/group with 4 technical replicates per donor). C6.25, C25: Curcumin (6.25, 25 μg/mL); F12.5, F50: Fumaric acid (12.5, 50 μg/mL); A12.5, A50: Apigenin (12.5, 50 μg/mL).

Cell metabolic activity was assessed using the CCK-8 assay (Figure 1a). None of the tested treatments caused a significant change in metabolic activity compared with the untreated control. Similarly, LDH activity was evaluated as an indicator of cytotoxicity, and no significant differences were observed between any of the treatment groups and the untreated control (Figure 1b). Overall, these findings indicate that apigenin, curcumin, and fumaric acid did not exert detectable cytotoxic effects or significantly alter cellular metabolic activity under the experimental conditions applied. These results support the suitability of the tested concentrations for subsequent evaluation of inflammatory and oxidative stress-related responses.

### 3.2 Determination of intracellular ROS levels

**Figure 2:**
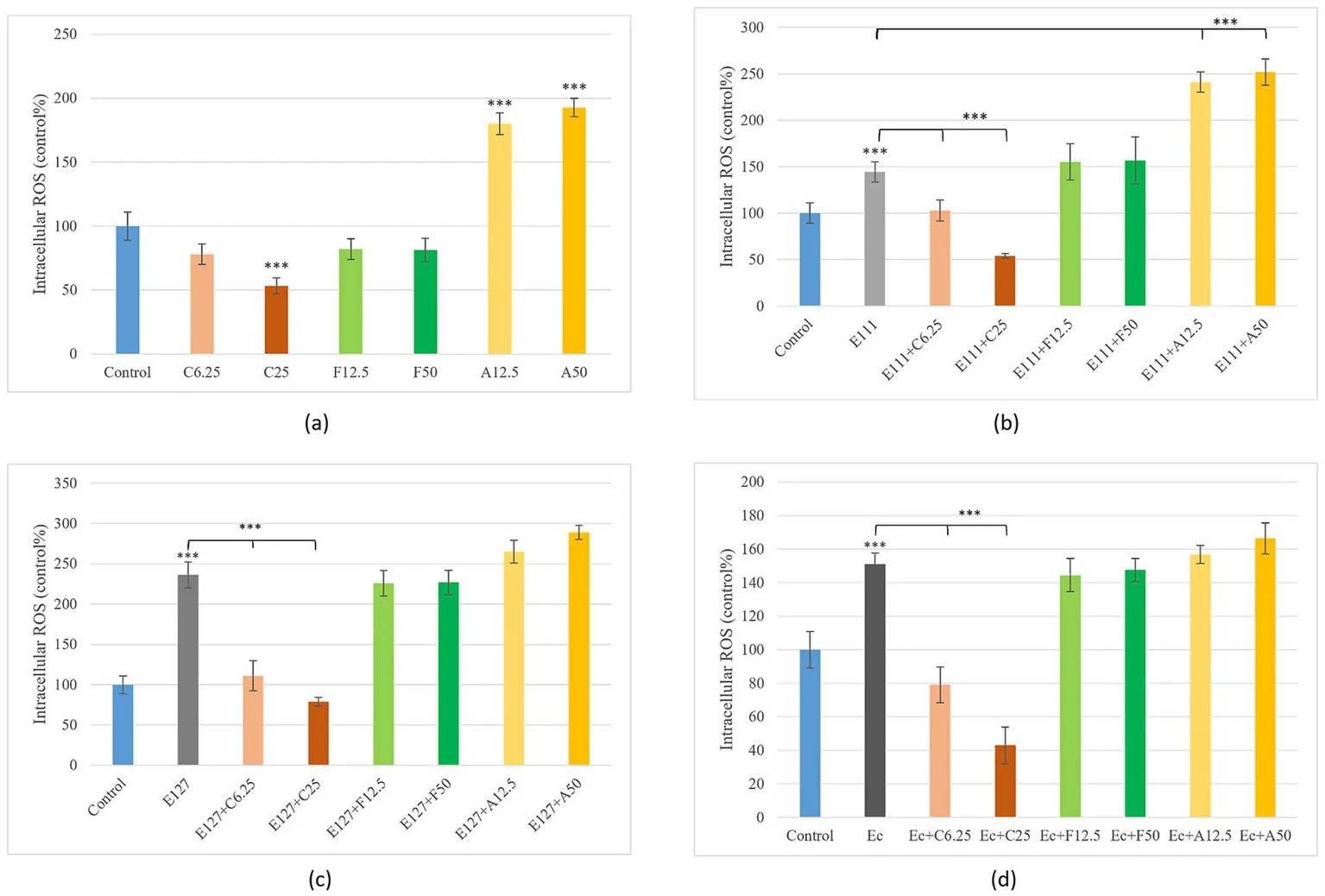
Effect of curcumin, fumaric acid, and apigenin treatments on intracellular ROS production in canine PBMC cultures measured using the DCFH-DA probe. **(a)** Antioxidant treatments without inflammatory stimulation (curcumin, fumaric acid, and apigenin) compared with the control group. **(b)** Effects of antioxidant treatments in the presence of *E. coli* O111 LPS (1 μg/mL). **(c)** Effects of antioxidant treatments in the presence of *E. coli* O127 LPS (1 μg/mL). **(d)** Effects of antioxidant treatments in the presence of enrofloxacin-treated *E. coli* isolate. The results are shown as mean values with corresponding standard deviations. The mean value of the control group was set at 100% (*n* = 3/group with 4 technical replicates per donor). C6.25, C25: curcumin 6.25 and 25 μg/mL; F12.5, F50: fumaric acid 12.5 and 50 μg/mL; A12.5, A50: apigenin 12.5 and 50 μg/mL; E111: *E. coli* O111 LPS (1 μg/mL); E127: *E. coli* O127 LPS (1 μg/mL); Ec: enrofloxacin-treated *E. coli* isolate. Significant difference: ^***^p < 0.001 compared to the control group or the corresponding LPS- or *E. coli*-treated groups.

The effects of curcumin, apigenin, and fumaric acid on intracellular ROS production were assessed under basal conditions and following stimulation with *E. coli* LPS or enrofloxacin-treated *E. coli* (Figure 2). Under unstimulated conditions, curcumin at 25 μg/mL significantly reduced intracellular ROS levels compared with the untreated control (*p* < 0.001). In contrast, apigenin at 12.5 and 50 μg/mL significantly increased basal ROS production compared with the control (*p* < 0.001 for both concentrations), whereas no significant differences were observed following treatment with fumaric acid (Figure 2a). Stimulation with E111 LPS significantly increased intracellular ROS production compared with the untreated control (*p* < 0.001) (Figure 2b). Curcumin at 6.25 and 25 μg/mL significantly attenuated the LPS-induced increase in ROS levels compared with the corresponding LPS-stimulated group *(p* < 0.001 for both concentrations). In contrast, apigenin at 12.5 and 50 μg/mL significantly increased ROS production compared with the LPS-stimulated group (*p* < 0.001 for both concentrations). Similarly, E127 LPS stimulation resulted in a significant increase in intracellular ROS production compared with the untreated control (*p* < 0.001) (Figure 2c). Treatment with curcumin at both tested concentrations significantly reduced ROS levels compared with the corresponding LPS-stimulated group (*p* < 0.001 for both concentrations).

Finally, stimulation with enrofloxacin-treated *E. coli* significantly increased intracellular ROS production compared with the untreated control (*p* < 0.001) (Figure 2d). Curcumin at both tested concentrations significantly attenuated ROS production compared with the corresponding *E. coli*-stimulated group (*p* < 0.001 for both concentrations).

### 3.3 Determination of extracellular H_2_O_2_ levels

**Figure 3:**
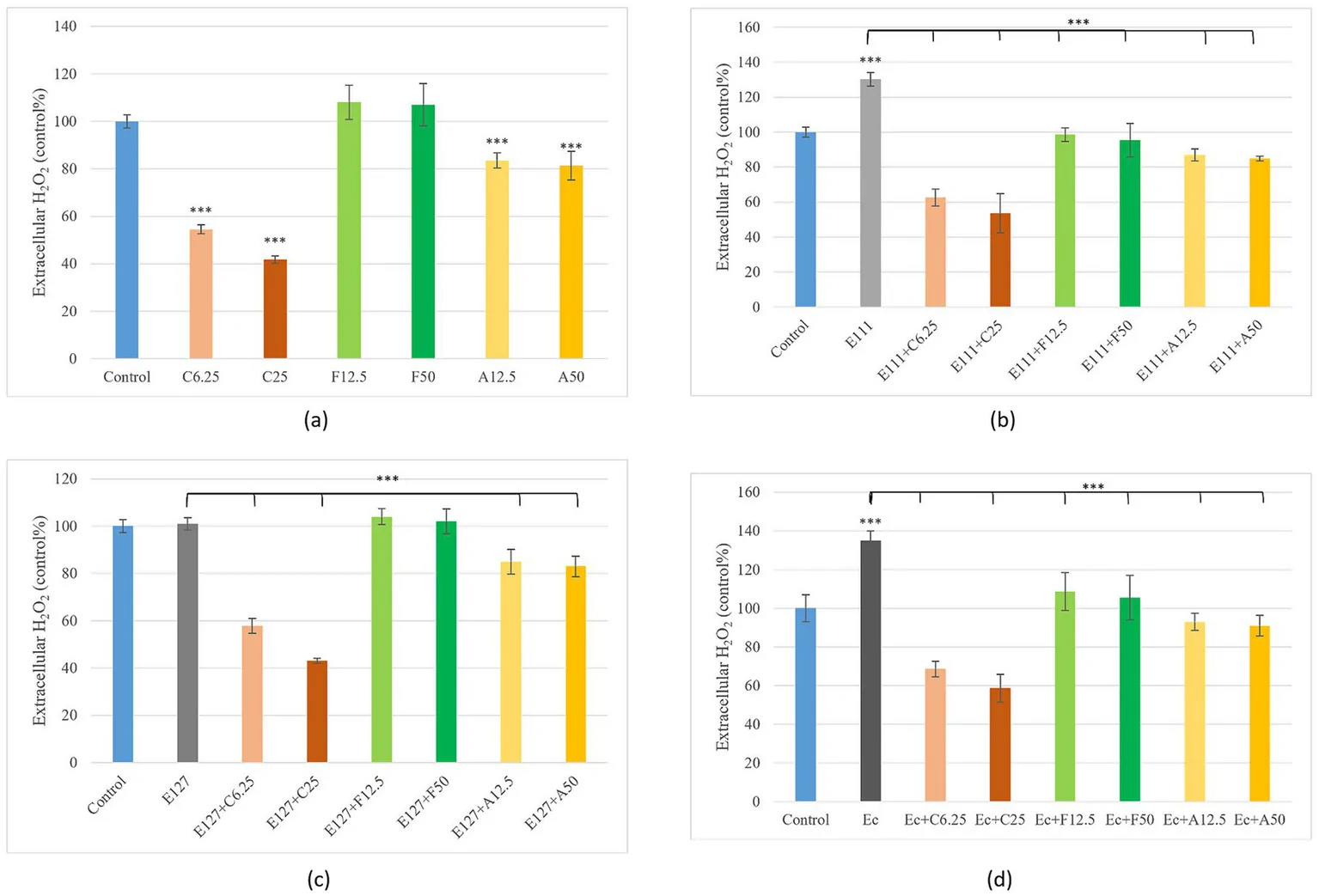
Effect of curcumin, fumaric acid, and apigenin treatments on extracellular H_2_O_2_ production in canine PBMC cultures measured using the Amplex Red probe. **(a)** Antioxidant treatments without inflammatory stimulation (curcumin, fumaric acid, and apigenin) compared with the control group. **(b)** Effects of antioxidant treatments in the presence of *E. coli* O111 LPS (1 μg/mL). **(c)** Effects of antioxidant treatments in the presence of *E. coli* O127 LPS (1 μg/mL). **(d)** Effects of antioxidant treatments in the presence of enrofloxacin-treated *E. coli* isolate. The results are shown as mean values with corresponding standard deviations. The mean value of the control group was set at 100% (*n* = 3/group with 4 technical replicates per donor). C6.25, C25: curcumin 6.25 and 25 μg/mL; F12.5, F50: fumaric acid 12.5 and 50 μg/mL; A12.5, A50: apigenin 12.5 and 50 μg/mL; E111: *E. coli* O111 LPS (1 μg/mL); E127: *E. coli* O127 LPS (1 μg/mL); Ec: enrofloxacin-treated *E. coli* isolate. Significant difference: ^***^p < 0.001 compared to the control group or the corresponding LPS- or *E. coli*-treated groups.

To further characterize the oxidative response and to complement the intracellular ROS measurements, extracellular H_2_O_2_ production was assessed using the Amplex Red assay (Figure 3). Under unstimulated conditions, curcumin and apigenin significantly reduced H_2_O_2_ production at both tested concentrations compared with the untreated control (*p* < 0.001 for all comparisons), whereas no significant effect was observed following treatment with fumaric acid (Figure 3a). Stimulation with E111 LPS significantly increased H_2_O_2_ production compared with the untreated control (Figure 3b). Apigenin, curcumin, or fumaric acid significantly reduced H_2_O_2_ production at both tested concentrations compared with the corresponding LPS-stimulated group (*p* < 0.001 for all comparisons). Following E127 LPS stimulation, no significant difference in H_2_O_2_ production was observed compared with the untreated control. However, treatment with curcumin and apigenin at both tested concentrations significantly reduced H_2_O_2_ production compared with the corresponding LPS-treated group (*p* < 0.001 for all comparisons) (Figure 3c). Stimulation with enrofloxacin-treated *E. coli* significantly increased H_2_O_2_ production compared with the untreated control (Figure 3d). Apigenin, curcumin, or fumaric acid at both tested concentrations significantly reduced H_2_O_2_ production compared with the corresponding *E. coli*-stimulated group (*p* < 0.001 for all comparisons).

### 3.4 Determination of TNF-α levels

**Figure 4:**
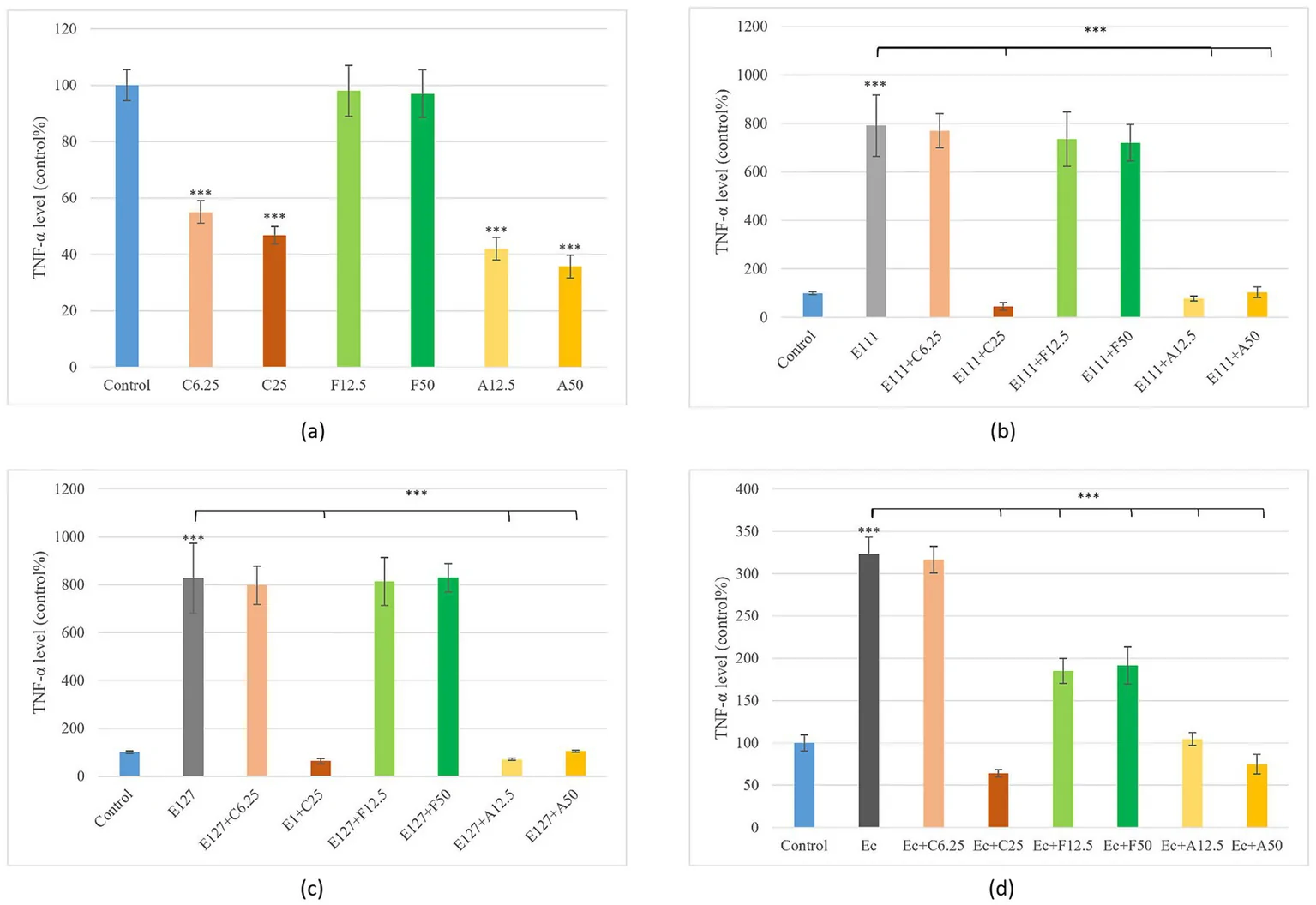
Effect of curcumin, fumaric acid, and apigenin treatments on TNF-α production in canine PBMC cultures. **(a)** Antioxidant treatments without inflammatory stimulation (curcumin, fumaric acid, and apigenin) compared with the control group. **(b)** Effects of antioxidant treatments in the presence of *E. coli* O111 LPS (1 μg/mL). **(c)** Effects of antioxidant treatments in the presence of *E. coli* O127 LPS (1 μg/mL). **(d)** Effects of antioxidant treatments in the presence of enrofloxacin-treated *E. coli* isolate. The results are shown as mean values with corresponding standard deviations. The mean value of the control group was set at 100% (*n* = 3/group with 4 technical replicates per donor). C6.25, C25: Curcumin 6.25 and 25 μg/mL; F12.5, F50: Fumaric acid 12.5 and 50 μg/mL; A12.5, A50: Apigenin 12.5 and 50 μg/mL; E111: *E. coli* O111 LPS (1 μg/mL); E127: *E. coli* O127 LPS (1 μg/mL); Ec: Enrofloxacin-treated *E. coli* isolate. Significant difference: ^***^p < 0.001 compared to the control group or the corresponding LPS- or *E. coli*-treated groups.

The effects of curcumin, apigenin, and fumaric acid on TNF-α production were assessed under basal conditions and following stimulation with *E. coli* LPS or enrofloxacin-treated *E. coli* (Figure 4). Under unstimulated conditions, curcumin and apigenin significantly reduced TNF-α levels at both tested concentrations compared with the untreated control (*p* < 0.001 for all comparisons), whereas fumaric acid did not significantly affect basal TNF-α production (Figure 4a). Stimulation with E111 LPS significantly increased TNF-α production compared with the untreated control (*p* < 0.001) (Figure 4b). Treatment with curcumin at 25 μg/mL and apigenin at both 12.5 and 50 μg/mL significantly reduced TNF-α production compared with the corresponding LPS-stimulated group (*p* < 0.001 for all comparisons). No significant reduction was observed following treatment with fumaric acid. A similar response pattern was observed following E127 LPS stimulation (Figure 4c). E127 LPS significantly increased TNF-α production compared with the untreated control (*p* < 0.001). Curcumin at 25 μg/mL and apigenin at both tested concentrations significantly reduced TNF-α production compared with the corresponding LPS-stimulated group (*p* < 0.001 for all comparisons), whereas fumaric acid did not produce a significant effect. Stimulation with enrofloxacin-treated *E. coli* also resulted in a significant increase in TNF-α production compared with the untreated control (*p* < 0.001) (Figure 4d). Under these conditions, curcumin at 25 μg/mL, apigenin at both tested concentrations, and fumaric acid at both tested concentrations significantly reduced TNF-α production compared with the corresponding *E. coli*-stimulated group (*p* < 0.001 for all comparisons).

### 3.5 Antimicrobial activity and interaction studies

The antimicrobial activity of apigenin, curcumin, and fumaric acid, as well as the MIC and MPC values of the reference antibiotics, was evaluated against four canine *E. coli* isolates (No. 340, 404, 863, and 865). The MIC and MPC values of the reference antibiotics varied among the isolates, with the most pronounced differences observed for gentamicin and enrofloxacin. The MIC and MPC values obtained for all isolates are presented in Table 1.

**Table 1:** Antimicrobial activity of apigenin, curcumin, and fumaric acid and antibiotics against canine *E. coli* isolates.

Isolate	Compound	MIC (μg/mL)	MPC (μg/mL)
340	Apigenin	>1024^*^	>1024^*^
	Curcumin	1,024	>1024^*^
	Fumaric acid	1,024	>1024^*^
	Amoxicillin–clavulanic acid	8	8
	Gentamicin	64	64
	Enrofloxacin	0.03125	64
404	Apigenin	>1024^*^	>1024^*^
	Curcumin	1,024	>1024^*^
	Fumaric acid	1,024	>1024^*^
	Amoxicillin–clavulanic acid	16	32
	Gentamicin	1	2
	Enrofloxacin	0.015625	0.03125
863	Apigenin	>1024^*^	>1024^*^
	Curcumin	1,024	>1024^*^
	Fumaric acid	1,024	>1024^*^
	Amoxicillin–clavulanic acid	8	16
	Gentamicin	2	2
	Enrofloxacin	0.03125	0.03125
865	Apigenin	>1024^*^	>1024^*^
	Curcumin	1,024	>1024^*^
	Fumaric acid	1,024	>1024^*^
	Amoxicillin–clavulanic acid	8	16
	Gentamicin	1	1
	Enrofloxacin	0.03125	128

^*^Values above 1,024 μg/mL should be interpreted cautiously because of limited aqueous solubility and compound precipitation.

MIC, minimum inhibitory concentration; MPC, mutant prevention concentration.

For apigenin, curcumin, and fumaric acid, MIC values were at or above the highest reliably assessable concentration of 1,024 μg/mL. Determination of MPC values was similarly limited by the poor aqueous solubility of the compounds, with increasing precipitation observed at higher concentrations. Therefore, concentrations exceeding 1,024 μg/mL were not considered reliable for antimicrobial activity assessment and were excluded from subsequent checkerboard analyses. Owing to these solubility limitations, checkerboard assays and FIC index determinations were performed only at concentrations up to 1,024 μg/mL. Concentrations exceeding this threshold were excluded from the interaction studies because precipitation could interfere with antimicrobial activity and compromise the interpretation of synergistic or antagonistic effects.

Apigenin exhibited indifferent interactions with all three tested antibiotics across all four *E. coli* isolates. Curcumin also showed predominantly indifferent interactions across the isolates. The only synergistic interaction observed for curcumin was with amoxicillin–clavulanic acid in isolate No. 863, at curcumin concentrations ranging from 32 to 256 μg/mL, where the MIC of amoxicillin–clavulanic acid decreased from 8 to 1 μg/mL. All other curcumin–antibiotic combinations showed indifferent interactions. Fumaric acid showed indifferent interactions with amoxicillin–clavulanic acid and gentamicin across all four isolates. In contrast, an antagonistic interaction between fumaric acid and enrofloxacin was consistently observed at fumaric acid concentrations of 512 and 1,024 μg/mL in all four *E. coli* isolates. Under these conditions, the MIC of enrofloxacin increased fourfold. Overall, the checkerboard analysis revealed predominantly indifferent interactions between the tested compounds and antibiotics, with a single synergistic interaction between curcumin and amoxicillin–clavulanic acid in isolate No. 863 and a consistent concentration-dependent antagonistic interaction between fumaric acid and enrofloxacin across all four isolates (Table 2).

**Table 2:** Interaction of apigenin, curcumin, and fumaric acid with antibiotics against canine *E. coli* isolates.

Isolate	Antioxidant	Antibiotic	Antioxidant concentration (μg/mL)	Interaction	Antibiotic MIC alone (μg/mL)	Antibiotic MIC in combination (μg/mL)
340	Apigenin	Amoxicillin–clavulanic acid	≤1,024	Indifferent	8	8
		Enrofloxacin	≤1,024	Indifferent	0.03125	0.03125
		Gentamicin	≤1,024	Indifferent	64	64
	Curcumin	Amoxicillin–clavulanic acid	≤1,024	Indifferent	8	8
		Enrofloxacin	≤1,024	Indifferent	0.03125	0.03125
		Gentamicin	≤1,024	Indifferent	64	64
	Fumaric acid	Amoxicillin–clavulanic acid	≤1,024	Indifferent	8	8
		Enrofloxacin	512–1,024	Antagonistic	0.03125	0.125
		Gentamicin	≤1,024	Indifferent	64	64
404	Apigenin	Amoxicillin–clavulanic acid	≤1,024	Indifferent	16	16
		Enrofloxacin	≤1,024	Indifferent	0.015625	0.015625
		Gentamicin	≤1,024	Indifferent	1	1
	Curcumin	Amoxicillin–clavulanic acid	≤1,024	Indifferent	16	16
		Enrofloxacin	≤1,024	Indifferent	0.015625	0.015625
		Gentamicin	≤1,024	Indifferent	1	1
	Fumaric acid	Amoxicillin–clavulanic acid	≤1,024	Indifferent	16	16
		Enrofloxacin	512–1,024	Antagonistic	0.015625	0.0625
		Gentamicin	≤1,024	Indifferent	1	1
863	Apigenin	Amoxicillin–clavulanic acid	≤1,024	Indifferent	8	8
		Enrofloxacin	≤1,024	Indifferent	0.03125	0.03125
		Gentamicin	≤1,024	Indifferent	2	2
	Curcumin	Amoxicillin–clavulanic acid	32–256	Synergistic	8	1
		Enrofloxacin	≤1,024	Indifferent	0.03125	0.03125
		Gentamicin	≤1,024	Indifferent	2	2
	Fumaric acid	Amoxicillin–clavulanic acid	≤1,024	Indifferent	8	8
		Enrofloxacin	512–1,024	Antagonistic	0.03125	0.125
		Gentamicin	≤1,024	Indifferent	2	2
865	Apigenin	Amoxicillin–clavulanic acid	≤1,024	Indifferent	8	8
		Enrofloxacin	≤1,024	Indifferent	0.03125	0.03125
		Gentamicin	≤1,024	Indifferent	1	1
	Curcumin	Amoxicillin–clavulanic acid	≤1,024	Indifferent	8	8
		Enrofloxacin	≤1,024	Indifferent	0.03125	0.03125
		Gentamicin	≤1,024	Indifferent	1	1
	Fumaric acid	Amoxicillin–clavulanic acid	≤1,024	Indifferent	8	8
		Enrofloxacin	512–1,024	Antagonistic	0.03125	0.125
		Gentamicin	≤1,024	Indifferent	1	1

MIC, Minimum inhibitory concentration; MPC, Mutant prevention concentration.

## 4 Discussion

In the canine gastrointestinal tract, a diverse and dynamic microbial community forms a complex ecosystem essential for maintaining intestinal and systemic homeostasis. In healthy individuals, the dominant bacterial phyla include Fusobacteria, Bacteroidetes, Firmicutes, Proteobacteria, and Actinobacteria (45). Disruption of this balance, referred to as dysbiosis, is a hallmark of CIE and is associated with both local intestinal pathology and systemic consequences. In CIE, impaired intestinal barrier integrity facilitates the translocation of bacterial products, particularly LPS, into the systemic circulation, contributing to persistent low-grade endotoxemia and chronic immune activation. In addition to sustaining local inflammation, endotoxemia has been associated with extraintestinal consequences affecting renal, cardiovascular, and neurological systems, emphasizing the importance of systemic immune modulation in disease management (6).

Natural bioactive compounds with antioxidant and immunomodulatory properties have therefore gained increasing attention as potential supportive therapeutic agents. Oxidative stress plays a key role in the pathophysiology of CIE, contributing to epithelial dysfunction, disruption of barrier integrity, and amplification of inflammatory signaling cascades (5). In the present study, curcumin, apigenin, and fumaric acid were investigated in an *in vitro* canine PBMC model under endotoxin- and bacteria-associated inflammatory conditions. PBMCs represent a clinically relevant model for evaluating systemic immune consequences of intestinal inflammation because circulating immune cells are directly exposed to bacterial products translocated across a compromised intestinal barrier (46). Although PBMC responses do not fully reproduce epithelial and mucosal immune interactions occurring *in vivo*, they provide valuable mechanistic insight into systemic inflammatory signaling associated with endotoxemia.

Stimulation with both LPS serotypes and enrofloxacin-treated *E. coli* was associated with changes in intracellular ROS production, H₂O₂ release, and TNF-α levels, although the magnitude and pattern of responses varied among the different stimuli. The two LPS serotypes were selected to assess whether the observed cellular responses were consistent across distinct *E. coli*-derived endotoxin preparations rather than being restricted to a single LPS serotype. In contrast, enrofloxacin-treated *E. coli* may have provided a more complex bacterial stimulus, potentially involving multiple bacterial components released or exposed following antibiotic treatment. Together, these measurements allowed the inflammatory and oxidative responses to the different experimental stimuli to be assessed from complementary perspectives. Previous studies have linked endotoxin-induced inflammatory responses to TLR4 activation (5). These mechanisms may contribute to the responses observed here, although they were not directly examined in the present study.

Assessment of metabolic activity and LDH release demonstrated that none of the investigated compounds significantly affected PBMC metabolic activity or LDH release under the tested experimental conditions. Previous studies have reported favorable effects of curcumin and related natural compounds on immune cell viability and cellular stress responses (47). Although the present findings do not demonstrate a significant cytoprotective effect of the investigated compounds, the absence of detectable cytotoxicity is important for the interpretation of the subsequent immunomodulatory findings, as the observed changes in inflammatory and oxidative stress-related parameters are unlikely to be attributable to nonspecific cellular damage.

Intracellular ROS, H_2_O_2_, and TNF-α measurements revealed compound- and stimulus-dependent effects, highlighting the complex regulation of oxidative and inflammatory responses in LPS- and bacteria-stimulated canine PBMCs. Curcumin showed the most consistent antioxidant-like pattern across the different experimental conditions. It reduced intracellular ROS following both E111 and E127 LPS stimulation and also reduced H_2_O_2_ production under conditions associated with increased oxidative responses. This concordance between the two complementary readouts supports an overall attenuation of the oxidative response by curcumin under the experimental conditions. These findings are consistent with previous reports describing antioxidant and anti-inflammatory properties of curcumin and its capacity to modulate cellular redox balance (5, 47). However, the present study did not directly investigate the molecular pathways underlying these effects; therefore, specific signaling mechanisms should not be inferred from the present findings.

The response to apigenin was more complex. Although apigenin increased intracellular ROS under basal conditions and following the lower-intensity E111 LPS stimulation, this effect was not observed when the oxidative stimulus was more pronounced. In parallel, Amplex Red measurements showed a significant reduction in H_2_O_2_ production following apigenin treatment under several experimental conditions. These apparently divergent effects may reflect differences in the oxidant species detected by the two assays. The DCFH-based intracellular ROS assay provides a broad measure of cellular oxidative activity rather than a specific measurement of H_2_O_2_, as DCFH oxidation can be influenced by several reactive species and cellular redox reactions (48). Therefore, an increase in DCF fluorescence does not necessarily imply a corresponding increase in H_2_O_2_ production. The present findings may thus indicate that apigenin affects different components of the cellular redox network in distinct ways.

The possibility of assay-related interference was specifically considered because of the intrinsic color and chemical properties of the investigated compounds. Cell-free controls demonstrated a mild, concentration-dependent effect of apigenin on the DCF-based signal, and the corresponding measurements were adjusted for this background. Nevertheless, the observed interference was limited and did not fully account for the cellular response. Since the cells were washed between compound exposure and DCF measurement, direct extracellular interference by residual apigenin should have been reduced; however, residual compound associated with the cells or intracellularly retained apigenin cannot be completely excluded as a contributing factor. This possibility, together with the known limitations of DCFH-based assays, warrants cautious interpretation of the apigenin-associated increase in intracellular ROS. Previous studies have also described context-dependent pro-oxidant effects of apigenin, suggesting that its redox activity may depend on cell type, concentration, and the prevailing oxidative environment (49). The stimulus-dependent nature of the apigenin response is particularly noteworthy. Its ROS-enhancing effect was apparent under basal conditions and at the lower oxidative stimulus induced by E111 LPS, whereas no additional increase was observed when the LPS-induced oxidative response was already substantially elevated. This pattern may suggest that the effect of apigenin is dependent on the cellular redox state and may become less apparent once the oxidative response reaches a higher level. However, this interpretation remains speculative and requires further mechanistic investigation.

A comparable stimulus-dependent pattern was observed for fumaric acid in the H_2_O_2_ measurements. Fumaric acid did not significantly alter H_2_O_2_ production under basal conditions or following E127 LPS stimulation, where no significant increase in H_2_O_2_ was detected relative to the control. In contrast, when the oxidative response was significantly elevated, as observed following E111 LPS or enrofloxacin-treated *E. coli* stimulation, fumaric acid significantly reduced H_2_O_2_ production. These findings suggest that the effect of fumaric acid may become more apparent under conditions of increased oxidative stress rather than under basal conditions.

The changes observed in oxidative stress-related parameters were accompanied by alterations in TNF-α production, providing an additional indication of the immunomodulatory effects of the tested compounds. TNF-α production was significantly increased following stimulation with both E111 and E127 LPS, as well as with enrofloxacin-treated *E. coli*, confirming activation of the inflammatory response in the PBMC model. Curcumin reduced TNF-α production under these inflammatory conditions, although the effect was most consistently observed at 25 μg/mL. Apigenin also reduced TNF-α production at both tested concentrations following LPS and bacteria-associated stimulation. Interestingly, this anti-inflammatory effect occurred despite the increase in intracellular ROS observed with apigenin under basal and lower-intensity E111 stimulation. This further supports the interpretation that intracellular DCF-derived ROS and inflammatory cytokine production represent related but distinct biological readouts and should not necessarily be expected to change in parallel.

Fumaric acid showed a more stimulus-dependent effect on TNF-α production. It did not significantly alter TNF-α levels under basal conditions or following E111 and E127 LPS stimulation, whereas it significantly reduced TNF-α production following stimulation with enrofloxacin-treated *E. coli*. Together with the H_2_O_2_ findings, this may indicate that the effects of fumaric acid become more apparent under specific inflammatory or oxidative conditions rather than during basal cellular states. Nevertheless, further studies would be required to determine whether these differences reflect stimulus-specific cellular responses or differences in the intensity and nature of the inflammatory challenge.

In addition to TNF-α, IL-1β, IL-2, and IL-6 were also assessed as potential inflammatory readouts. However, their concentrations in the culture supernatants were below the reliable detection range under the applied experimental conditions and therefore did not permit meaningful quantitative analysis. Consequently, TNF-α was used as the principal cytokine readout in the present study. The selective detectability of TNF-α may reflect differences in cytokine production kinetics or magnitude in canine PBMCs under the experimental conditions used.

Taken together, the complementary assessment of intracellular ROS, H₂O₂, and TNF-α demonstrates that the effects of apigenin, curcumin, and fumaric acid were dependent on both the compound and the nature and intensity of the inflammatory stimulus. Curcumin showed the most consistent reduction across oxidative and inflammatory readouts, whereas apigenin displayed a more complex redox profile characterized by increased DCF-derived intracellular ROS under selected conditions but reduced H_2_O_2_ and TNF-α production. Fumaric acid showed comparatively limited effects under basal conditions but exerted measurable effects under selected stimulated conditions. These findings support the potential of the tested compounds to modulate inflammatory and oxidative responses relevant to canine endotoxemia, while also emphasizing the importance of using complementary assays when evaluating redox-active compounds.

The antimicrobial evaluation demonstrated that apigenin, curcumin and fumaric acid exhibited MIC and MPC values substantially exceeding concentrations considered biologically achievable *in vivo*, suggesting limited direct antibacterial efficacy under physiologically relevant conditions (50). However, this finding should not necessarily diminish their therapeutic relevance, as their primary contribution may lie in modulation of host inflammatory responses rather than direct pathogen elimination. Accordingly, these compounds may be better regarded as supportive adjunctive agents rather than alternatives to conventional antimicrobial therapy.

Interestingly, curcumin demonstrated a synergistic interaction with amoxicillin–clavulanic acid specifically against the canine *E. coli* isolate No. 863, reducing the MIC of the antibiotic from 8 μg/mL to 1 μg/mL. No synergistic interaction was observed for this combination against the other tested isolates (Nos. 340, 404, and 865). Previous studies have attributed the antibacterial activity of curcumin partly to effects on bacterial membrane integrity and permeability (51). Such mechanisms may potentially contribute to interactions with conventional antibiotics; however, the mechanism underlying the isolate-specific synergistic interaction observed here was not investigated (52). Such membrane-destabilizing effects may enhance the penetration and intracellular activity of β-lactam antibiotics, providing a plausible explanation for the reduction in amoxicillin–clavulanic acid MIC observed in the present study. Combination studies with enrofloxacin revealed predominantly neutral interactions at concentrations corresponding to those used in PBMC experiments, indicating that the tested compounds are unlikely to compromise antibacterial activity within biologically relevant concentration ranges. Although previous studies reported synergistic antibacterial interactions between curcumin and enrofloxacin against selected *E. coli* strains, particularly enterotoxigenic isolates (53), such effects were not observed under the present experimental conditions. These discrepancies may be attributable to differences in bacterial strains and their susceptibility profiles, as well as variations in the concentrations at which interactions were evaluated. Notably, antagonistic interactions with enrofloxacin were observed only at fumaric acid concentrations above 512 μg/mL. A plausible explanation is the acidification of the culture medium at elevated fumaric acid concentrations, resulting in decreased pH and consequently reduced enrofloxacin activity. Fluoroquinolone efficacy has been reported to decline under acidic conditions, partly due to altered bacterial uptake and reduced antimicrobial activity, which may account for the antagonistic effects observed at the highest fumaric acid concentrations (54).

Taken together, the present findings highlight curcumin as the most consistent antioxidant and anti-inflammatory compound among those investigated, while apigenin demonstrated context-dependent immunomodulatory effects characterized by simultaneous anti-inflammatory activity and differential modulation of oxidative responses. Fumaric acid exerted moderate but biologically relevant immunomodulatory effects that appeared more strongly linked to cytokine suppression than direct ROS regulation. Collectively, these findings suggest that these natural compounds may modulate inflammatory responses and intracellular ROS production in this *in vitro* canine PBMC model. Whether these effects translate into reduced cellular oxidative stress or oxidative damage requires further investigation *in vivo*.

Several limitations should nevertheless be acknowledged. First, although the use of PBMCs from three independent donor animals increased the biological replication of the study, the cells were obtained from healthy dogs and therefore may not fully reflect the altered immune state of dogs affected by CIE, septicemia, or endotoxemia. In addition, PBMCs represent a peripheral immune cell population and do not recapitulate the complex cellular and tissue-specific environment of the intestinal tract. Consequently, the present model is more appropriately considered an *in vitro* model of systemic inflammatory and oxidative responses associated with endotoxemia rather than a direct model of intestinal immune responses in CIE. It does not account for intestinal epithelial barrier function, microbiota interactions, local intestinal immune cell populations, or tissue-specific inflammatory processes. The concentrations used in the PBMC experiments were selected based on previous experimental work and published in vitro data; however, their correspondence to achievable plasma or tissue concentrations in dogs has not been established. Therefore, the present findings should not be interpreted as evidence that the tested concentrations are pharmacologically attainable *in vivo*. Second, the present study focused primarily on short-term inflammatory and oxidative stress-related endpoints, including intracellular ROS, H_2_O_2_, and TNF-α. Although additional cytokines, including IL-1β, IL-2, and IL-6, were assessed, their concentrations in the culture supernatants were below the reliable detection range under the applied experimental conditions. Additional inflammatory markers, transcriptional analyses, and mechanistic studies could therefore provide further insight into the cellular pathways underlying the observed responses. Furthermore, the use of isolated PBMCs and LPS- or bacteria-stimulated conditions cannot fully reproduce the multifactorial nature of CIE or naturally occurring endotoxemia. Finally, the poor aqueous solubility of apigenin and curcumin at higher concentrations resulted in partial or complete precipitation above 1,024 μg/mL, limiting the reliable determination of MIC, MPC, and FIC values within this concentration range. Accordingly, the antimicrobial findings at higher nominal concentrations should be interpreted with caution, as the effective dissolved concentrations may have been lower than the nominal concentrations.

In conclusion, curcumin, apigenin, and fumaric acid produced distinct, stimulus-dependent changes in inflammatory and oxidative readouts in canine PBMCs. Curcumin reduced intracellular ROS levels and TNF-α production under several experimental conditions, apigenin displayed context-dependent modulation of oxidative responses despite marked TNF-α suppression, while fumaric acid showed more limited, stimulus-dependent effects, particularly on inflammatory responses. The present findings demonstrate compound- and stimulus-dependent modulation of intracellular ROS, H_2_O_2_, and TNF-α responses in canine PBMCs under defined inflammatory conditions. These findings support further investigation of these compounds as candidate modulators of systemic inflammatory and oxidative responses associated with endotoxemia. Whether these effects are relevant to naturally occurring CIE, including intestinal barrier function or clinical outcomes, requires validation in disease-relevant models and *in vivo* studies.

## Data Availability

The raw data supporting the conclusions of this article will be made available by the authors, without undue reservation.
